# The Various Scoring Systems in Pediatric Intensive Care Units: A Prospective Observational Study

**DOI:** 10.7759/cureus.39679

**Published:** 2023-05-30

**Authors:** Jyotsna, Rakesh Kumar, Shambhavi Sharan, Sunil Kishore, Jayant Prakash

**Affiliations:** 1 Pediatrics, Indira Gandhi Institute of Medical Sciences, Patna, IND

**Keywords:** sofa, prism, pim, pelod, mortality

## Abstract

Background: The discrimination power of the pediatric risk of mortality (PRISM), pediatric index of mortality (PIM), sequential organ failure assessment (SOFA), and pediatric logistic organ dysfunction (PELOD) may not always be true for countries such as India due to differences in factors from those nations where these scoring systems were validated. Therefore, this study was undertaken to determine and compare severity, course of illness, and outcomes in critically ill children admitted to the pediatric intensive care unit (PICU) using different scoring systems such as PRISM 4, PIM 3, PELOD 2, and the pediatric sequential organ failure assessment (pSOFA ) score, and to analyze the clinical spectrum and demographic profile of children admitted to the PICU.

Materials and method: This was a prospective, single-center, observational study conducted in the PICU of the Indira Gandhi Institute of Medical Science, Patna, India, over two years. Two hundred children in the age group of one month to 14 years admitted to the PICU were recruited into the study. Prognostic scoring systems, including PRISM4 and PIM3, were used to compare the outcome, mortality, and length of PICU stay, whereas PELODS and pSOFA were descriptive scores that assessed the multiorgan dysfunction. A correlation between the different scoring systems and the outcome was determined.

Results: The majority of children (26.5%, n=53) were one to three years of age. The maximum number of patients was male (66.5%, n=133). Renal complications were the predominant admission diagnosis in 19% (n=38) of children. The mortality rate was found to be 18.5%. The mortality was most common in infants <1 year of age (n=11, 29.73%) and those of the male sex (n=22, 59.46%). A significant correlation was found between length of stay and mortality (p<0.00001). A significant positive correlation was observed between mortality and PRISM 4, PIM 3, PELOD 2, and pSOFA scores on the first day of admission (p<0.00001). The pSOFA and PELOD2 showed better discrimination power (area under the curve (AUC): 0.77 and 0.74, respectively).

Conclusion: The study concluded that the pSOFA and PELOD2 scores are reliable predictors of mortality in critically ill children.

## Introduction

The current period in the history of the pediatric intensive care unit (PICU) is marked by an advanced and intense system. Pediatric intensive care units are presently very expensive and complex. Pediatric critical care medicine arose in the 1960s and has expanded dramatically. It has made major advances in the areas of traumatic brain injury, lung injury, sepsis, and postoperative care, and it has led to new intensive care-related medical and societal responsibilities. The key purpose of the PICU is to reduce mortality. Those who are critically ill and deemed to be at a higher mortality risk are closely monitored and given the care necessary to accomplish this [1,2]. According to the severity of their sickness and the patient group under research, between 6.4% and 10.3% of critically ill patients were reported to die in the ICU [3]. Irrespective of the underlying sickness, insufficient data is known about the precise reasons for mortality. Understanding these variables will aid in the analytical evaluation of these patients and also help pinpoint areas of therapy and research required to improve their short- and long-term outcomes. Pediatric patients who are in critical condition exhibit significant deviations from the body's normal balance. The drift of the physiological variables from the normal range can be used to estimate these variations, and the deviations of these drifting variables can be used to create scores [4,5].

Prognostic scoring systems were developed in the 1980s and have since been improved and validated to assist in assessing illness severity for risk stratification in terms of required resources, stratifying patients before randomization in clinical trials, improving quality assessment as well as cost-benefit analysis, comparing intra and interinstitutional outcome and survival, and facilitating clinical decision making [6-8]. The early grading systems were designed for adults and weren’t as effective with kids. Finally, scores that were specifically developed and upgraded for children were presented. Some of them allow the survival probability to be calculated as a function of the final score. The pediatric risk of mortality (PRISM) score and its subsequent iterations, the PRISM III and PRISM IV scores, the pediatric index of mortality (PIM) score, the PIM2 and PIM3 scores, and the pediatric logistic organ dysfunction score (PELOD), followed by the PELOD 2 score, are examples of current scores that are particularly appropriate for children [9].

Mortality, length of stay, functional outcome, and organ dysfunction are the PICU outcomes that are most frequently measured [10]. There is a lack of information about the comparison of different prognostic and descriptive scores in terms of outcome, mortality, and length of PICU stay in patients admitted to the PICU. Therefore, the current study was undertaken to compare different prognostic scores (PRISM 4 and PIM 3) in terms of outcome, mortality, and length of PICU stay for patients in the PICU, and both PELODS and pediatric sequential organ failure assessment (pSOFA) were used to assess the descriptive score for multiorgan dysfunction.

## Materials and methods

This was a prospective, single-center, observational study conducted in the PICU of the Indira Gandhi Institute of Medical Sciences, Patna, India from November 2020 to November 2022 (approval no. 1956/IEC/IGIMS/2020). Children in the age group of one month up to 14 years admitted to the PICU were considered eligible. Patients with an ICU stay of <24 hours and admitted for elective procedures like intravenous immunoglobulin (IVIG) administration, post-hemodialysis observation, and central line insertion, were excluded.

Two to three patients are admitted daily to our PICU. Considering this rate of admission, the period total admission will be 1540. Based on a 5% margin of error and a 95% confidence interval (CI) with a response distribution of 18%, the sample size is 198.

Two hundred children were recruited into the study protocol after obtaining written informed consent from either the parents or the guardians. At the time of admission baseline demographic data, and clinical information including a detailed history and examination were documented and laboratory investigations were conducted as per the ICU protocol. A provisional diagnosis was made based on the initial workup. The clinical course during the hospital stay and the outcome of the case was also documented.

Prognostic scoring systems including PRISM4 and PIM3 were used to compare the outcome, mortality, and length of PICU stay whereas PELODS and pSOFA were descriptive scores that assessed the multiorgan dysfunction. Variables of PIM3 and PRISM4 were collected within one hour and four hours of PICU admission, respectively. Data were interpreted using the online mortality calculator on Collaborative Pediatric Critical Care Research Network (CPCCRN). Data for PELOD 2 was collected on days 1, 2, 5, 8, 12, 16, and 18. The pSOFA scoring was done on days 1, 2, 4, 7, and 14. A correlation between the different scoring systems and the outcome was determined.

Statistical analysis

Data were collected in approved proforma and entered into a Microsoft Excel (Microsoft Corp., Redmond, WA, USA) sheet. Data were analyzed using the SPSS version 20 (IBM Corp., Armonk, NY, USA) software. Continuous variables were expressed in terms of mean±SD whereas, categorical variables were expressed in percentage and frequency. Statistical significance was tested using the Student t-test for quantitative values, the chi-square test was used for qualitative values, and other tests of significance were used depending on the results. A p<0.05 was considered statistically significant.

## Results

The majority of pediatric patients (26.5%, n=53) were aged between one and three years. The maximum number of patients was male (66.5%, n=133). Renal complications were the predominant admission diagnosis in 19% (n=38) of patients (Table 1).

**Table 1:** Distribution of subjects according to different variables CNS: Central nervous system, CVS: Chorionic villus sampling, MODS: Multiorgan dysfunction syndrome, GIT: Gastrointestinal tract

Variables	Subcategories	Frequency (n)	Percentage (%)
Age (years)	<1	40	20
	1-3	53	26.5
	4-6	36	18
	7-10	42	21
	11-14	29	14.5
Sex	Male	133	66.5
	Female	67	33.5
Diagnosis	Respiratory	24	12
	CNS	28	14
	CVS	7	3.5
	Sepsis/infection	28	14
	MODS	14	7
	GIT	28	14
	Hematoma	12	6
	Oncological	6	3
	Metabolic	5	2.5
	Poisoning	2	1
	Renal	38	19
	Other	8	4

Thirty-seven (18.5%) patients died, whereas 163 (81.5%) survived. The mortality rate was found to be 18.5%. The incidence of mortality was most common in infants <1 year of age (n=11, 29.73%) and males (n=22, 59.46%). The mean duration of hospital stays for all patients was 8.32 days. The mean duration of hospital stay in survived and non-survived patients was 7.7±7.70 days and 8.57±5.49 days, respectively. A significant correlation was found between length of stay and mortality (p<0.00001).

The PRISM 4, PIM 3, pSOFA, and PELOD 2 scores were significantly higher in patients who expired than in patients who survived. A significant positive correlation was observed between mortality and PIM 3, PRISM 4, PELOD 2, and pSOFA scores on the first day of admission (p<0.00001). Both pSOFA and PELOD2 showed better discrimination power (area under the curve (AUC): 0.77, 0.74, respectively) (Table 2).

**Table 2:** Correlation between scores and outcomes PRISM: Pediatric risk of mortality, PIM: Pediatric index of mortality, PELOD: Pediatric logistic organ dysfunction, pSOFA: Pediatric sequential organ failure assessment, AUC: Area under the curve

Scoring systems	Expired (mean±SD)	Survived (mean±SD)	p-value	AUC
PRISM 4	20.29±8.48	6.75±8.30	<0.00001	0.71
PIM 3	37.19±0.21	3.80±0.20	<0.00001	0.68
PELOD 2	11.27±4.90	2.71±4.72	<0.00001	0.74
pSOFA	13.45±5.28	4.42±5.13	<0.00001	0.77

## Discussion

The use of scoring systems in the PICU may facilitate early decision-making and appropriate management [11,12]. The PRISM, PIM, pSOFA, and PELOD are well-known scoring systems used for determining short-term outcomes. However, its discriminatory powers may not always be true for developing countries such as India due to the varying patient profiles and limitations of resources and experienced healthcare workers. Also, we get more cases of infectious origin in comparison to the origin countries of the scoring system, where patients with genetic disorders and trauma are more common. Moreover, protein-energy malnutrition is still a common problem in our patients. Therefore, this study was undertaken to determine and compare severity, course of illness, and outcomes in critically ill patients admitted to the PICU using the PRISM 4, PIM 3, PELOD 2, and pSOFA scoring systems and to analyze the clinical spectrum and demographic profile of patients admitted in the PICU.

The mortality rate in the study population was 18.5%. The age group associated with mortality was <1 year followed by one to three years, four to six years, seven to 10 years, and 11-14 years. Around 59.46% of the patients who expired were male. These findings suggested that the mortality rate is higher in infants, particularly in male infants. Various other studies have suggested rates of mortality ranging from 21.66% to 57% [ 13-16]. The difference in the rate of mortality may be due to the difference in the type of study, study setting, inclusion criteria, geographical area, etc. This also suggests higher mortality rates in infants <1 year of age.

In this study, there were 38 renal cases with three deaths, 28 sepsis/infection cases with six deaths, 28 gastrointestinal tract (GIT) cases with seven deaths, 28 central nervous system (CNS) cases with one death, 24 respiratory cases with three deaths, 14 cases with 12 deaths, seven chorionic villus sampling (CVS) cases with one death, 12 hematological cases with one death, six oncological cases with one death, and five metabolic cases with one death. No mortality was seen in two cases of poisoning and eight cases with other causes. These findings suggest that the existence of multi-organ dysfunction (MODS) on the day of admission was associated with higher mortality which correlates to the studies by Typpo et al. and Costa et al [17-19]. In the study by Rady et al., the most common admission diagnosis was a respiratory problem followed by CNS and CVS problems. They reported a high proportion of mortality in cases with sepsis, MODS, and neurological disease [3].

In the present study, PRISM 4, PIM 3, PELOD 2, and pSOFA scores were seen to be significantly increased in patients that expired than in patients who survived. Also, a significantly positive correlation was observed between mortality and PRISM 4, PIM 3, PELOD 2, and pSOFA scores on the first day of admission (p<0.00001). These findings are comparable with the studies conducted by Rady et al., Patki et al., and El‐Nawawy [3,13,19]. These findings suggest that these scoring systems can satisfactorily differentiate survivors from non-survivors. In this study, we found that the discrimination power of pSOFA and PELOD 2 was better than other scoring modalities, which are again similar to the study of Rady et al. and Lalitha et al. [3,20]. Furthermore, the study by Baloch et al. showed better discrimination power of pSOFA compared to PRISM 3 [15]. However, a meta-analysis conducted by Shen et al. suggested higher discrimination power of PRISM 3/4 compared to PELOD 2 and PIM 3 [21]. The difference in the results may be due to the difference in the study population, cause of admission, etc.

Although all scoring systems showed increased scores in non-surviving patients compared to surviving patients, the present study showed that the pSOFA score had a strong ability to predict mortality among the admitted patients compared to the PELOD score. We found that the increase in pSOFA score from day 1 to day 14, increases the chance of mortality. These findings are comparable with the study of Khajeh et al, and Lalitha et al. [22,20].

## Conclusions

The PRISM 4, PIM 3, PELOD 2, and pSOFA scores were found to be significantly increased in non-surviving pediatric patients compared to pediatric patients who survived. Both pSOFA and PELOD 2 showed better discrimination power compared to other scoring systems and can be reliable predictors of mortality in critically ill children. These scoring systems are well-known in developed countries, necessitating their validation in patients in developing countries. The PRISM 4 and PRISM 3 are predictors of mortality, while PELOD 2 and pSOFA are primarily day-to-day assessments of critically ill pediatric patients. This study found these scoring systems to be equally valid in developing countries as well. The study has some limitations; due to the type of study, the quality of the data recorded could intimidate the validity of the findings. To obtain more reliable and exact results, further studies with a larger sample size are required.
